# Quality Assessment of *Mycobacterium tuberculosis* Genotyping in a Large Laboratory Network

**DOI:** 10.3201/eid0811.020401

**Published:** 2002-11

**Authors:** Christopher R. Braden, Jack T. Crawford, Barbara A. Schable

**Affiliations:** * Centers for Disease Control and Prevention, Atlanta, Georgia, USA

**Keywords:** *Mycobacterium tuberculosis*, DNA fingerprinting, quality assessment, molecular epidemiology

## Abstract

Quality assessment exercises were conducted to evaluate the reproducibility of IS*6110* DNA fingerprinting performed by eight laboratories in the National Tuberculosis Genotyping and Surveillance Network. Three panels, each with 8 to 16 isolates, were typed at all laboratories, resulting in 280 images. When the pattern obtained by the majority for each isolate was used as the standard, exact matches were obtained for 73% of patterns; 90% and 97% of patterns matched within one- and two-band differences, respectively. A second approach involved retyping of randomly selected isolates at the Centers for Disease Control and Prevention. Retyping was done for 8–19 isolates per laboratory (76 total). Paired images matched exactly for 54% of isolates and within one and two band differences, 78% and 93%, respectively. We evaluated reasons for mismatching. We also evaluated the reproducibility of spoligotyping using a test panel of 13 isolates; a discrepancy of 1 in 91 results was noted.

A proposed standard methodology for *Mycobacterium tuberculosis* genotyping was published in 1993 (1). This methodology, restriction fragment length polymorphism (RFLP)–based analysis using IS*6110* as a marker, has been adopted by laboratories worldwide for studying the molecular epidemiology of tuberculosis. Although other methods have been introduced, IS*6110* fingerprinting provides the highest specificity and remains the most effective and consistent genotyping technique for *M. tuberculosis*. Standardization of this technique promises the best results for direct comparison of large numbers of genotype patterns obtained in different laboratories (2–4). Common *M. tuberculosis* genotype patterns from distant populations may be sought to determine geographic mobility of related strains or to identify clonal ancestry in evolutionary genetics. Alternatively, the ability to divide the genotyping workload among several laboratories may allow more complete genotyping for larger host populations.

In 1996, the National Tuberculosis Genotyping and Surveillance Network adopted the standard IS*6110* fingerprinting method for primary genotyping (5). To test the proficiency of laboratories and determine the reproducibility of this genotyping technique in a large network of laboratories, the Centers for Disease Control and Prevention (CDC) instituted quality assessment exercises for the seven genotyping network laboratories. These exercises included sending panels of isolates (from CDC stocks) to all laboratories and retyping (at CDC) a sample of isolates for which an IS*6110* RFLP pattern was previously submitted to the central database. Through these quality assessment exercises, we identified common causes for mismatched patterns and determined the frequency of mismatch occurrences among the laboratories.

## Methods

### Genotyping

IS*6110* DNA fingerprinting was performed according to standard methods (1,5). The genotyping network protocol provided standardization of the procedure among laboratories, including use of the same size standards, gel sizes, electrophoresis run conditions, and IS*6110* probes. Size standards were applied in outside and middle lanes (three total) for each gel; standards internal to each lane were not used. Gel electrophoresis equipment and reagent components, such as agarose, were not specified in the protocol and varied among the laboratories. Spoligotyping was performed by the standard procedure (6).

### Isolate Panel Quality Assessment Exercise

Three test panels were sent to each of seven genotyping network laboratories. CDC also genotyped the isolates in all panels, so a total of eight laboratories participated in the overall assessment. For two test panels, 16 and 13 isolates selected from CDC stocks were subcultured in 7H9 broth, then onto Lowenstein-Jensen slants, and sent to the seven genotyping network laboratories for genotyping. Occasionally, cultures became contaminated, or a technical mishap occurred; therefore, not every laboratory submitted an image for every isolate. For one panel, DNA was purified from eight isolates at CDC, and aliquots of DNA were sent to all laboratories. With few exceptions, each laboratory ran each panel of isolates on a single gel. The resulting images were digitized and analyzed by using BioImage Whole Band Analyzer software, version 3.4 (BioImage, Ann Arbor, MI) with standardized analysis parameters (5). The match parameter included a possible 2.5% deviation in calculated molecular weights among compared bands, a parameter setting recommended by BioImage developers, experienced users in genotyping network, and other experts. Digital images of the edited autoradiograms indicating operator determination of band placement were transmitted electronically to the CDC network coordinator for comparison. We conducted these exercises during years 1, 2, and 5 of the 5-year project.

### Isolate Retyping Quality Assessment Exercise

We selected recent genotypes submitted by the genotyping network laboratories to the central fingerprint database from a spreadsheet, basing selection on pattern band numbers to standardize the complexity of patterns distribution among laboratories. Isolates were routinely frozen and stored at –70°C at each participating laboratory. Selected isolates were recultured and sent to CDC, where they were retyped according to genotyping network protocol.

### Spoligotyping Quality Assessment Exercise

The 13 isolates in the third panel were also spoligotyped at six genotyping network laboratories and CDC. Each laboratory analyzed the resulting patterns and provided a digital result for comparison.

### Matching Outcome

For each isolate in the panels, we defined a reference pattern as the pattern that matched exactly by the greatest number of laboratories; in 62% of isolates, at least five of eight results matched exactly. Each isolate pattern was compared to the reference pattern. For isolates retyped at CDC, each isolate pattern from the genotyping network laboratory was compared with the CDC pattern. Neither pattern was assumed to be the correct result. Misalignment between a band in one pattern and the same band in a compared pattern was considered a one-band mismatch. We placed the outcomes in one of the following categories: an exact match (i.e., same number and size of bands), an exact match with the exception of one band (match ±1 band), an exact match with the exception of two bands (match ±2 bands), and no match (three or more bands different). We included only computer-derived comparisons of original, blinded pattern determinations; we did not include any judgments after the computer match in the analysis. Reasons for nonexact matches were categorized as the addition or omission of one or more bands in one pattern compared with the other, mismatch of individual bands in compared patterns, and a shift up or down in one pattern compared with the other.

## Results

### Isolate Panel Quality Assessment Exercise

Tests by eight laboratories of three panels (8–16 isolates each) resulted in 280 images from 37 isolates. Overall, an exact match was achieved for 73% of all patterns (range by isolate, 33% to 100%; range by panel, 66% to 85%); 90% matched ±1 band (range by isolate, 63% to 100%; range by panel, 85% to 98%); and 97% matched ±2 bands (range by isolate, 86% to 100%; range by panel, 96% to 98%) (Table 1).

**Table 1:** Percent of restriction fragment length polymorphism images matching reference pattern^a^ for all isolates in quality assessment panels

	Panel 1 % (16 isolates, 124 images)	Panel 2 % (8 isolates, 53 images)	Panel 3 % (13 isolates, 103 images)	All panels % (37 isolates, 280 images)
Match	73	85	66	73
Match ±1^b^	91	98	85	90
Match ±2^b^	98	98	96	97

^a^Reference pattern was the pattern that matched exactly in the greatest number of laboratories. ^b^Match ±1, exact match with the exception of one band; match ±2, exact match with the exception of two bands.

No single laboratory achieved exact matches to the reference pattern for all isolates. One laboratory matched ±1 band for all isolates and three laboratories ±2 bands for all isolates. All laboratories matched the reference pattern ±2 bands for at least 90% of isolates (Table 2).

**Table 2:** Number and percent of restriction fragment length polymorphism images from each laboratory matching reference pattern in quality assessment panels

Laboratory	No. of patterns submitted by laboratory	Match^a^ (%)	Match ±1^b^ (%)	Match ±2^c^ (%)
1	36	27 (75)	33 (92)	36 (100)
2	39	29 (74)	35 (90)	38 (97)
3	35	27 (77)	35 (100)	35 (100)
4	38	33 (87)	37 (97)	38 (100)
5	38	33 (87)	35 (92)	36 (95)
6	34	22 (65)	28 (82)	33 (97)
7	27	13 (48)	25 (93)	26 (96)
8	33	20 (61)	25 (76)	30 (91)

^a^ Number of patterns submitted by laboratory that matched exactly the reference pattern. ^b^ Match ±1, exact match with the exception of one band. ^c^Match ±2, exact match with the exception of two bands.

**Figure 1:**
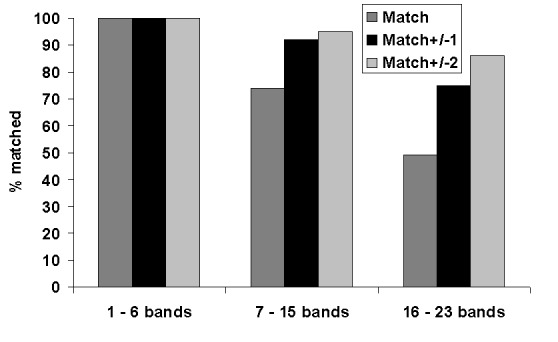
Quality assessment panel match results shown by number of bands in patterns.

Patterns with a low number of bands (1–6 bands) constituted 20% of patterns; 55% of patterns contained a midrange number of bands (7–15), and 25% of patterns had a high number of bands (16–23). Figure 1 shows the matching results for the three categories of patterns. For low-band number patterns, 100% of images matched exactly. For midband number patterns, 74% matched exactly; and for high–band number patterns, 49% matched exactly. Within limits of ±2 bands, 95% of mid–band number images matched, and 86% of high–band number images matched.

**Figure 2:**
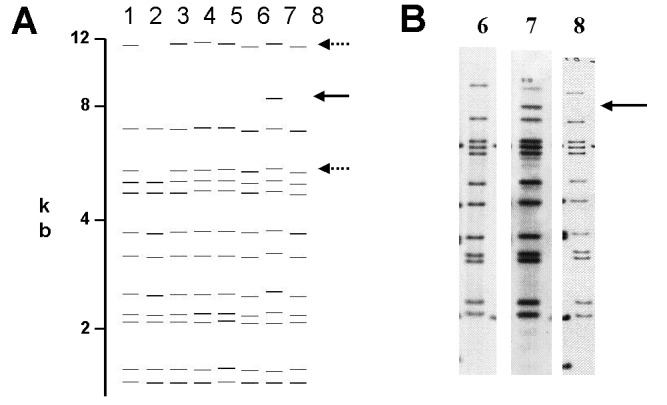
A) Computer-derived IS*6110* restriction fragment length polymorphism patterns from eight laboratories for one isolate. Addition or omission of bands is demonstrated. B) Autoradiogram images demonstrating the addition of IS*6110* band in restriction fragment length polymorphism pattern in one subpopulation of *Mycobacterium tuberculosis* isolates used in the quality assessment exercise.

**Figure 3:**
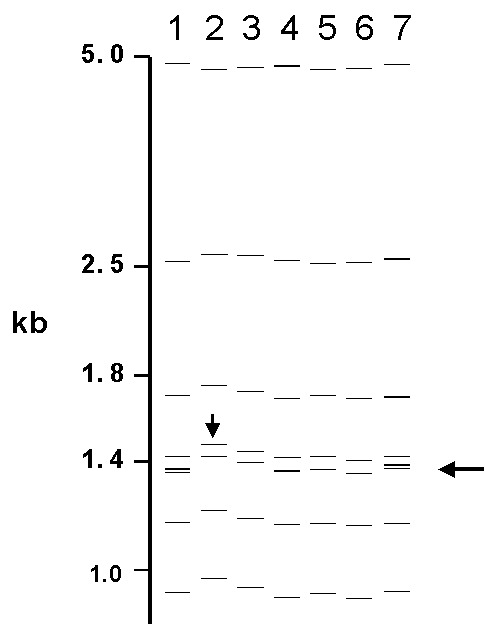
Computer-derived IS*6110* restriction fragment length polymorphism patterns from seven laboratories for one isolate. Misidentified doublet and shifted patterns are demonstrated.

Of the 76 images that did not match exactly the reference pattern for each strain, 41 (54%) showed addition or omission of one or more bands compared to others. Figure 2A shows the normalized, computer-generated lane maps of patterns obtained with one isolate. Although most bands in all of the patterns matched with very small deviations in size, the pattern in lane 2 is missing two bands, and the pattern in lane 7 has one additional band. Figure 2B shows the original IS*6110* RFLP autoradiogram image of the lane with the additional band and two representative images from other laboratories. The extra band is clearly present in the middle image from lane 7 and absent on the others, indicating a true difference in patterns derived from the same isolate. A specific class of discrepancies included the omission of high molecular weight (>10 kb) bands as shown in lane 2 of Figure 2A, which accounted for 10 (13%) of all mismatches. High molecular weight fragments also caused discrepancies in size determinations, especially when they were larger than the size standard (i.e., >15 kb). Figure 3 shows differences in band determination. Two laboratories (lanes 1 and 7) identified a doublet, which was called a single band in the other five laboratories. This type of band misidentification accounted for 31 (76%) of the 41 patterns that did not match the reference patterns because of the omission of one or more bands.

The second most common reason for nonmatches was a misalignment (16%) of individual bands (i.e., images contained a band, but it did not fall within 2.5% of the molecular weight of the band in other images). In additional cases, mismatching was caused by shifts in the pattern (5%), as shown in Figure 3, lane 2. Multiple factors for nonmatching (12%) accounted for the remainder of mismatches. Of images that did not match the reference pattern, 28% matched exactly the image from at least one other laboratory.

### Isolate Retyping Quality Assessment Exercise

**Figure 4:**
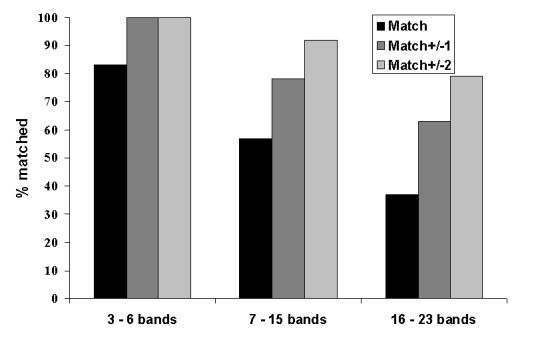
Quality assessment retyping match results by number of bands in the IS*6110* restriction fragment length polymorphism patterns.

Seventy-six distinct isolates with genotype images previously submitted to the central database were retyped. Of these, 8% of patterns had 3–6 bands, 67% of patterns had 7–15 bands, and 25% of patterns had 16–23 bands. Overall, 54% of retyped images matched exactly the original submitted image (range by laboratory, 25% to 80%); 77% of image pairs matched ±1 band (range by laboratory, 60% to 100%); and 93% matched ±2 bands (range by laboratory, 67% to 100%). Figure 4 shows the results stratified by pattern band number. Thirty-seven percent of high–band number pattern pairs matched exactly; the proportion improved to 89% with a match of ±2 bands. The presence or absence of bands in one pattern compared with the other accounted for 37% of nonmatching pattern pairs, multiple reasons accounted for 23%, individual band mismatches accounted for 20%, omission of a high molecular band accounted for 11%, and whole pattern shifts accounted for 9%.

### Spoligotyping Results

Spoligotyping results were compared for 13 isolates typed at the seven laboratories. Subjective judgments regarding the hybridization patterns were made at the laboratories, and only the final digital results were compared. Identical results were obtained for 90 of the 91 spoligotypes. The one differing result occurred because three consecutive oligonucleotide spacers were absent.

## Discussion

We used quality assessment exercises with the laboratories in the genotyping network to evaluate the reproducibility and interlaboratory variability of IS*6110* DNA fingerprinting. The genotyping network project included analyses of large databases of pattern images, with the use of computer-assisted matching algorithms to identify genotype clusters. This method is more complex and difficult than a visual interpretation of a small number of pattern images. The results of the quality assessment exercises suggest that exact computer-identified matches in images produced by the eight laboratories were reproducible for patterns with a small number of bands but not reliably reproducible for complex patterns with large numbers of bands.

Some differences observed were not the result of varying interpretation of the patterns. In Figure 2A, the discrepant band is clearly present in one sample and absent in the others. This discrepancy may have been caused by the presence of two subpopulations of bacteria that emerged upon subculture in the laboratories, a situation often recognized by the presence of faint bands in images (7). However, this discrepancy also occurred in the panel consisting of aliquots of DNA samples provided by CDC. A true difference in the populations of the isolate typed may account for the discrepant results of one laboratory in the spoligotyping exercise; the outlier pattern had three consecutive oligonucleotide spacers missing compared with the others, possibly because of a deletion in the direct repeat sequence of the genome of that *M. tuberculosis* sample. These observations suggest that IS*6110* replication and deletions in the direct repeat sequence may occur within subcultured populations of *M. tuberculosis* during a short period of time. However, none of these observed changes were independently verified by genomic mapping. Absence of high-molecular-weight bands was also common and was likely the result of poor transfer of DNA during the blotting procedure. Absence of high-molecular-weight bands can also result from degraded DNA samples.

Another cause for the differences in patterns relates to variability in band identification, specifically the determination of intense or wide bands as one, two, or even three fragments and discrimination among closely spaced bands (Figure 3). This determination often requires operator judgment and editing in pattern analysis and thus is prone to operator-dependent bias. The exposure times of autoradiograms and the intensity of bands may have influenced this determination and the outcome of the matching procedure. Table 2 shows this type of subjectivity for laboratory 7, where heavy or wide bands tended to be overcalled as multiple bands. The resulting rate of exact matches for laboratory 7 was 48%; the rate jumped to 93% for matches ±1 band.

Some mismatches resulted from small variations in sizing of bands. In many cases, patterns appeared the same on visual inspection, but specific bands fell outside of the 2.5% deviation in calculated molecular weight allowed for matching. Two phenomena are represented. Uneven heating during gel electrophoresis yields variations in mobility in different lanes, which results in miscalculation of the fragment sizes in comparison to the external standards that could be up to four lanes distant. This problem can be reduced by including internal lane standards that allow normalization of individual lanes. However, this approach requires alignment of the images from the two separate probes and can introduce other errors. An unanticipated problem occurred in the computerized matching process when images were slightly misaligned (Figure 3). With two closely spaced bands, the analysis algorithm occasionally matched the upper band in one image to the lower band in the other image, leaving the remaining bands unmatched. This situation could occur despite the fact that the correct band match was within the 2.5% deviation limit. This type of mismatch is readily detected by visual comparison of the patterns.

Advantages and disadvantages exist in either increasing or decreasing the allowable deviation in band size in the matching process. Decreasing the allowable deviation would exacerbate some of the problems seen in this exercise concerning the difficulties in accurately sizing bands. Increasing the percent deviation allowed may have alleviated some observed mismatches. However, the risk of false matches of different patterns increases with a more liberal allowable deviation in band sizes in the matching process.

The problem of mismatches owing to size variations was magnified for high-molecular-weight fragments. Because of the logarithmic scale in sizing fragments, small differences in migration of large fragments result in large differences in molecular weight size calculation. Some fragments fall outside the range of the size standard (i.e., >15 kb), requiring extrapolation and causing additional inaccuracy.

In a number of instances, whole pattern shifts led to mismatching of many bands, which was typically the result of curvature across the lanes in the gel or distortion of the gel during the blotting process. Occasionally, a shift in the pattern resulted from overloading the sample DNA into the gel. These problems were apparent from observation of the image; such gels should have been rejected and run again. Pattern shifting was a greater problem during routine compilation of the genotyping network database in which images of single lanes, rather than entire gel images, were submitted. Software modifications for computerized matching may help decrease mismatching on the basis of whole pattern shifts.

The reproducibility and interlaboratory variability of IS*6110* fingerprinting in this quality assessment exercise does not necessarily represent the usual methods for pattern analysis and cluster identification for the genotyping network databases. In this exercise, results of computer matching after blind pattern editing were final. In actuality, pattern editing and cluster determination in the genotyping network were iterative processes. During normal analysis, the database manager recognized some of the problems that may occur, such as shifts in mobility; additional analysis and sample rerunning were then required to clarify the relationship of patterns. In some instances, images were reviewed, and easily reconcilable band placement was edited on the basis of definitive information about epidemiologic links among patients. The process is usually referred to as computer-assisted matching, and the database continually changed with updated pattern analysis. Thus, the outcome of the fluid process of pattern analyses and cluster determination was more accurate than the results of this exercise suggest. In addition, prospective cluster analyses and investigations were conducted at each genotyping network laboratory for patients in their respective sentinel surveillance sites; therefore, interlaboratory variability was limited to retrospective analyses of clustering of the combined database. Nonetheless, the amount of variability and nonreproducibility shown during the quality assessment is substantial and should be reflected in the interpretation of genotyping network results. Given this limitation among the genotyping network laboratories, which were experienced and well standardized, the ability to share RFLP DNA fingerprint images among laboratories that do not have strict standardization may be even more limited by interlaboratory variability.

This quality assessment exercise demonstrates the overall difficulty of combining and analyzing DNA fingerprint images from multiple laboratories. Although RFLP methodology has shown great discriminatory capacity and has been the most effective genotyping method for *M. tuberculosis* and many other pathogens, newer DNA sequence-based genotype methods should allow seamless computerization and objective analysis of results that would bypass many of the limitations described in this study. The potential benefits of these new methods are demonstrated by the near perfect reproducibility we obtained with spoligotyping. Because spoligotyping does not possess the discriminatory power needed to generally replace IS*6110* RFLP (8), a combination of spoligotyping and newer variable number tandem repeats assays (9) may provide adequate discrimination for most purposes, with IS*6110* RFLP reserved for resolution of selected sets of clustered isolates.
